# ALDEFLUOR activity, ALDH isoforms, and their clinical significance in cancers

**DOI:** 10.1080/14756366.2023.2166035

**Published:** 2023-01-18

**Authors:** Jiang-Jie Duan, Jiao Cai, Lei Gao, Shi-Cang Yu

**Affiliations:** a Department of Stem Cell and Regenerative Medicine, Southwest Hospital; Third Military Medical University (Army Medical University), Chongqing, China; b International Joint Research Center for Precision Biotherapy, Ministry of Science and Technology, Chongqing, China; c Institute of Pathology and Southwest Cancer Center, Southwest Hospital, Chongqing, China; d Ministry of Education, Key Laboratory of Cancer Immunopathology, Chongqing, China; e Department of Hematology, Xinqiao Hospital; Third Medical University (Army Medical University), Chongqing, China; f Jin-feng Laboratory, Chongqing, China

**Keywords:** ALDH, ALDEFLUOR, tumour, stem cell, inhibitor

## Abstract

High aldehyde dehydrogenase (ALDH) activity is a metabolic feature of adult stem cells and various cancer stem cells (CSCs). The ALDEFLUOR system is currently the most commonly used method for evaluating ALDH enzyme activity in viable cells. This system is applied extensively in the isolation of normal stem cells and CSCs from heterogeneous cell populations. For many years, ALDH1A1 has been considered the most important subtype among the 19 ALDH family members in determining ALDEFLUOR activity. However, in recent years, studies of many types of normal and tumour tissues have demonstrated that other ALDH subtypes can also significantly influence ALDEFLUOR activity. In this article, we briefly review the relationships between various members of the ALDH family and ALDEFLUOR activity. The clinical significance of these ALDH isoforms in different cancers and possible directions for future studies are also summarised.

## Introduction

The aldehyde dehydrogenase (ALDH) superfamily is the most important aldehyde-metabolizing enzyme system in the body and belongs to the group of NAD(P)^+^-dependent enzymes. The human ALDH superfamily includes 11 families^1^^,^^2^ and a total of 19 members: ALDH1s (1A1, 1A2, 1A3, 1B1, 1L1, and 1L2), ALDH2, ALDH3s (3A1, 3A2, 3B1, and 3B2), ALDH4A1, ALDH5A1, ALDH6A1, ALDH7A1, ALDH8A1, ALDH9A1, ALDH16A1, and ALDH18A1^1^^,^^3^. The genes encoding these enzymes are located on different chromosomal segments and are named based on their evolutionary relationships and on the amino acid homologies of the encoding proteins^4^. All enzyme subtypes in the ALDH family have similar structures, and most are homodimers or homotetramers. Each monomer contains 3 structural domains: a catalytic domain, a cofactor-binding domain, and an oligomerization domain. This family of enzymes plays important roles in embryo formation, development, cell proliferation, and differentiation^3^. Its main functions include the followings: (1) Participation in retinoic acid (RA) synthesis: ALDH1As and ALDH8A1 participate in the second step of RA synthesis, in which all-trans or 9-cis retinal is oxidised to produce RA^5^. ALDH1A1 mainly oxidises all-trans and 9-cis retinal into RA^6^^,^^7^, and ALDH1A3 mainly oxidises all-trans retinal^5^^,^^8^. The catalytic effects of ALDH1A1 on these 2 reactions are equivalent^6^^,^^7^; ALDH1A2 displays higher catalytic efficiency for all-trans retinal^9^, and ALDH8A1 almost exclusively catalyses 9-cis retinal^5^^,^^10^^,^^11^. (2) Detoxification: ALDHs can irreversibly oxidise endogenous and exogenous aldehydes into the corresponding acids with the participation of NADP^+^, thereby performing detoxification functions and helping maintain normal cell homeostasis and body functions^4^^,^^12^. (3) Participation in resistance to chemotherapeutic drugs (especially drugs derived from aldehyde intermediates): ALDH oxidises cyclophosphamide intermediate products into non-toxic carboxyphosphamide, thereby increasing the resistance of tumour cells to cyclophosphamide^13^^,^^14^. (4) ALDH4A1 can also reduce cellular levels of reactive oxygen species (ROS), thereby protecting the cells from ROS injury^15^.

The ALDH family participates in physiological processes mainly involved in the development of normal tissues and in cell metabolism^16^. For instance, ALDH1A1 is the most studied subtype and is extensively expressed in normal human tissues, including the brain, thyroid gland, lung, mammary gland, liver, pancreas, kidney, colon, bone, prostate, ovary, and cervix. Notably, ALDH1A1 expression was found to be closely associated with the occurrence, development, and therapeutic resistance of tumours and was considered a marker of cancer stem cells (CSCs)^16^. Consequently, targeting CSCs through interference with ALDHs is considered a novel strategy for the chemical prevention and treatment of tumours.

## History of ALDH activity detection

Traditional methods for the detection of ALDH subtypes include western blotting, reverse transcription–polymerase chain reaction (RT–PCR), spectrophotometry for the detection of enzyme activities, and immunohistochemistry (IHC)^17^. However, none of the above methods can be used to measure ALDH activity in viable cells^18^^,^^19^. By applying the ALDEFLUOR system, in which BODIPY-aminoacetaldehyde (BAAA) was used as the ALDH substrate and N,N-diethylaminobenzaldehyde (DEAB) as a negative control, researchers successfully isolated SSC^lo^/ALDH^br^ cells from human umbilical cord blood, and 40–90% of the isolated cells were CD34^+^CD38^lo/–^ haematopoietic stem cells (HSCs)^20^. The ALDEFLUOR system has the following advantages: (1) it can detect human stem/progenitor cells of multiple lineages, including haematopoietic, mammary gland, endothelial, and neural stem/progenitor cells as well as mesenchymal stem cells; (2) it is applicable to the isolation of stem/progenitor cells of other species and human CSCs; and (3) it can be used with cryopreserved cell samples. Thus, ALDEFLUOR has become the most commonly used method for detecting ALDH enzyme activity in viable cells and for the isolation of stem/progenitor cells (http://www.stemcell.com).

## Using ALDEFLUOR as a stem cell marker

ALDEFLUOR was originally used for the identification and sorting of haematopoietic stem/progenitor cells^20^^,^^21^. Subsequently, this method was used to label stem cells in other normal tissues, including neural stem cells, adipose-derived adult stem cells^22^, and muscle precursor cells^23^. In addition, ALDEFLUOR was gradually extended to the isolation of stem cells from acute myeloid leukaemia (AML) and solid tumours, including breast cancer^24^, oral squamous cell carcinoma (OSCC)^25^, sarcoma^26^, oesophageal squamous cell carcinoma^27^^,^^28^, oesophageal adenocarcinoma^29^, gastric cancer^30^^,^^31^, colorectal cancer^32–34^, head and neck squamous cell carcinoma^35^, thyroid cancer^36^, lung cancer^37–39^, cholangiocarcinoma (CHOL)^40^, hepatocellular carcinoma^41^^,^^42^, pancreatic cancer^43–45^, osteosarcoma^46–48^, prostate cancer^49^^,^^50^, bladder cancer^51–53^, glioblastoma^54^, melanoma^55^, ovarian cancer^56–58^, cervical cancer^59^^,^^60^ and multiple myeloma^61^. Therefore, the ALDEFLUOR technique has become an important tool in CSCs research.

## Pending controversy

Although the ALDEFLUOR detection and sorting system has been extensively applied in the field of stem cell/CSCs research, some important scientific questions remain to be answered. The most striking question is which enzyme or group of enzymes among the 19 members of the ALDH family determines the ALDEFLUOR-positive rate. Initially, the activity and positive rate of ALDEFLUOR were thought to be primarily determined by ALDH1A1^62^. However, 9 of the 19 ALDH isoforms (ALDH1A1, ALDH1A2, ALDH1A3, ALDH1B1, ALDH2, ALDH3A1, ALDH3A2, ALDH3B1, and ALDH5A1) were potentially involved in ALDEFLUOR activity^63^. The results also implied that DEAB inhibits various ALDH isoforms to different extents except for ALDH3B1 and ALDH5A1^63^^,^^64^. Furthermore, the results of in-depth studies suggested that ALDH isoforms exhibited differential expression patterns in various cancer types^63^^,^^65^^,^^66^.

### Haematopoietic system

**Figure 1.**
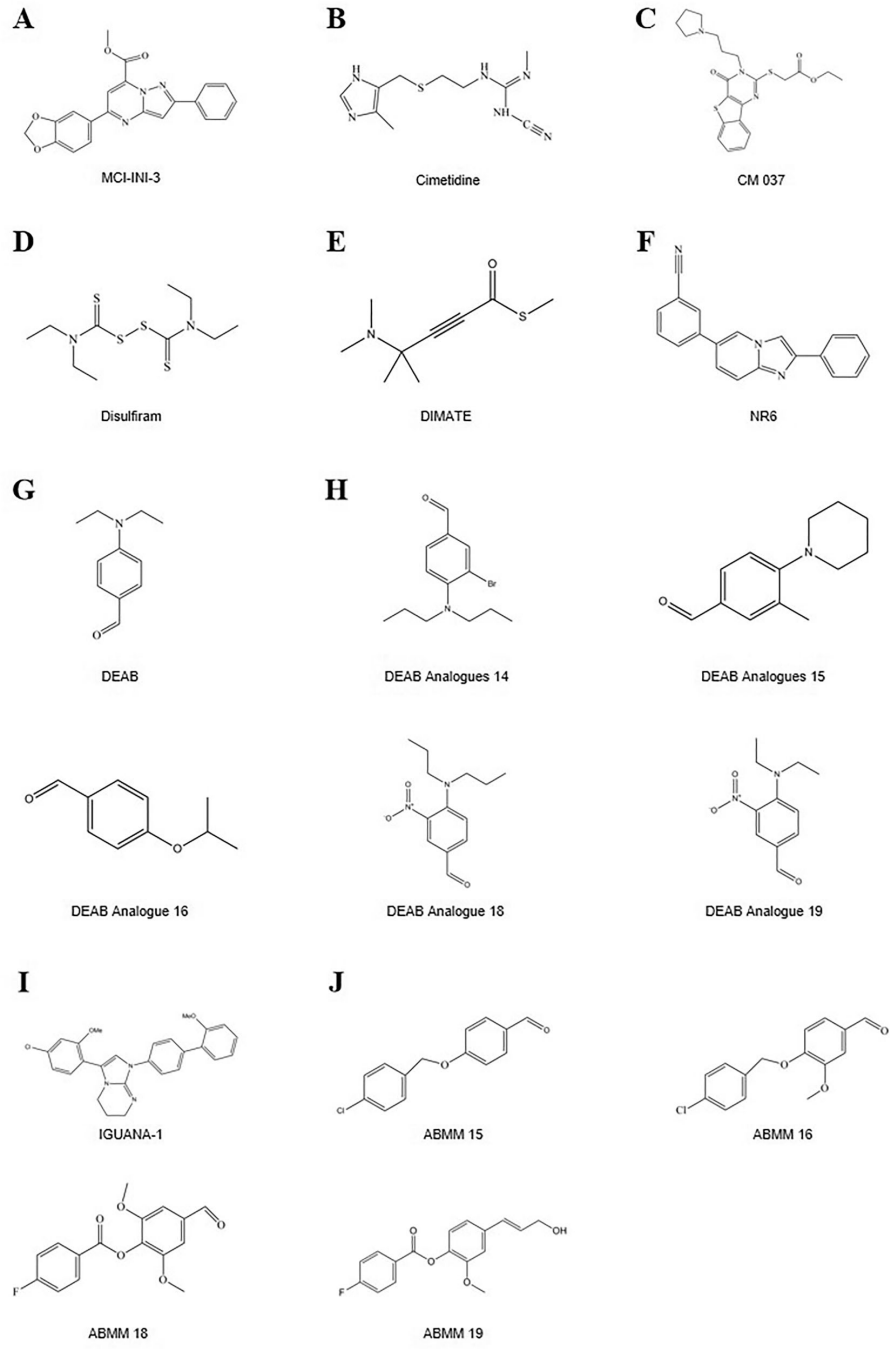
Chemical structures of ALDH inhibitors.

Accumulating evidence has indicated that ALDH might be a potential marker for HSCs and involved in leukaemia development^67^. ALDH1A1 was found to be most highly expressed in both murine and human HSCs and immature progenitors^68^. Moreover, studies have demonstrated that ALDH1A1 and ALDH3A1 are involved in the metabolism of ROS and reactive aldehydes in HSCs^69^^,^^70^, which may play important roles in HSC biology and leukaemia transformation^67^. With both ALDH1A1 and ALDH3A1 deletion, NUP98-HOXA10 homeodomain fusion protein can promote the development of leukaemia with B220^+^ and varied levels of CD11b, while NUP98-HOXA10 homeodomain fusion protein alone induces only *in vitro* expansion of HSCs without malignant transformation^71^, suggesting the important role of ALDH1A1 and ALDH3A1 in leukaemia initiation^67^^,^^72^. Additional evidence indicated that ALDH1A1 expression induced by cytokines in bone marrow cells can result in increased resistance to 4-hydroperoxycyclophosphamide (4-HC) in AML^73^, and suppression of ALDH1A1 can sensitise leukaemia cells to 4-HC^74^. Notably, inhibition of ALDH1A1 with all-trans RA was found to be a promising approach in AML by inducing the differentiation of leukaemia stem cells (LSCs)^75^. Disulfiram an inhibitor of ALDH1A1 (Figure 1), may offer a new treatment strategy by selectively eradicating LSCs through the simultaneous induction of ROS-JNK and inhibition of NF-kB and Nrf2^76^^,^^77^ and overcoming bortezomib and cytarabine resistance by inducing apoptosis and proteasome inhibition in AML^78^. The ALDH1A1 inhibitor dimethyl ampal thiolester (DIMATE) was also shown to be a drug candidate by specifically targeting LSCs in AML^79^ (Figure 1). In a study of multiple myeloma, ALDH + cells isolated by ALDEFLUOR were found to have stem cell-like characteristics. With overexpression of ALDH1A1 and ALDH2 in NCI-H929 cells, although ALDH activity detected by ALDEFLUOR assay is associated with the expression of both ALDH1A1 and ALDH2, only ALDH1A1 plays a pivotal role in maintaining the stemness of ALDH^+^cells^61^. Taken together, these findings indicated the therapeutic potential of targeting ALDH1A1 in the clinical management of AML patients^70^.

Previous studies have indicated that ALDEFLUOR^+^ cells account for approximately 1% of the total number of cells in bone marrow, mobilised peripheral blood cells, and umbilical cord blood^80^^,^^81^. These cells displayed the characteristics of HSCs, and the major enzyme subtype that determined the ALDEFLUOR-positive rate was ALDH1A1^81^. However, recent studies have reported that ALDH1A1 is not the dominant expression subtype in mouse HSCs and that the expression levels of ALDH subtypes in mouse HSCs follow the order ALDH9A1 > ALDH2 > ALDH1A1 ≥ ALDH3A2 > ALDH1A7^62^. In addition, upon knockout of ALDH1A1, ALDEFLUOR activity in mouse bone marrow stem cells and HSCs (CD150^+^CD41^−^CD48^−^Sca1^+^c-kit^+^) did not change significantly. However, if both ALDH1A1 and ALDH3A1 were knocked out in adult mouse bone marrow cells, the number of bone marrow stem cells and HSCs decreased significantly^68^^,^^69^^,^^82^. These results suggest that in addition to ALDH1A1, other ALDH subtypes, such as ALDH3A1, ALDH9A1, ADLH1A2, and ALDH7A1, might also influence ALDEFLUOR activity in HSCs^62^.

Similarly, TaqMan low-density array analysis of ALDEFLUOR^+^ and ALDEFLUOR^–^ populations of the K562 cell line reveal that in addition to the ALDH1 subfamily, K562 ALDEFLUOR^+^ cells also express the ALDH3, ALDH5, ALDH6, ALDH7, and ALDH8 subtypes^17^.

### Mammary gland

Recent efforts have suggested that ALDH1A1 is regulated at the posttranslational level by the Notch signalling pathway in breast cancer, which modulates ALDH1A1 acetylation through the induction of SIRT2 expression and therefore activates ALDH1A1 to promote tumorigenesis and tumour growth^83^. High ALDH1A1 activity was associated with a poor prognosis for breast cancer patients^24^. Breast CSCs, which exhibit high ALDH1A1 activity and high CD44 expression, were shown to be more sensitive to chemotherapy or radiation after pre-incubation with DEAB^84^. In vitro and in vivo treatment with all-trans RA has been reported to induce differentiation and chemotherapy sensitivity of resistant and metastatic breast CSCs, which were selected as CD44^+^/CD24^−/low^/ALDEFLUOR^+^ cells after long-term low-dose fractionated radiation treatment in MCF7/C6 cells^85^. Moreover, evidence has demonstrated that NANOG signalling induces enhanced ALDH1A3 activity through activation of the Notch1 and AKT pathways, which in turn stimulates DNA double-strand break repair capacity and confers radio resistance to breast cancer cell lines^86^. These results provide evidence for the selective suppression of ALDH1 isozymes (ALDH1A1 and ALDH1A3) as promising therapeutic targets in breast cancer^87^.

ALDEFLUOR^+^ cells isolated from normal mammary epithelial cells accounted for approximately 8% of the total number of cells and displayed mammary stem cell characteristics, including sphere formation ability, colony formation ability, and multidirectional differentiation^24^. IHC analysis further indicated that this ALDEFLUOR^+^ cell population mainly expressed ALDH1A1^24^ and was distributed in luminal areas of the gland, consistent with the localisation and presumed quantity of stem cells^88^. In addition, ALDEFLUOR^+^ mammary epithelial cells participate in duct formation and can be differentiated into uncommitted, luminal epithelial, and myoepithelial cells, whereas almost all ALDEFLUOR^–^ mammary epithelial cells produce luminal epithelial cells^24^. However, the results obtained by Rexer et al. indicated that ALDH1A3 determines the enzyme activity of ALDH in mouse mammary tissue and that it has a leading role in the synthesis of RA^89^. Furthermore, the normal mammary epithelial cell line MTSV1.7 expressed *ALDH1A3* mRNA and did not express *ALDH1A1* and *ALDH1A2* mRNAs^89^.

Viewpoints differ regarding the dominant ALDH subtype determining ALDEFLUOR activity in breast cancer. Deng et al. studied 15 breast cancer cell lines (BT-474, BT-483, BT-20, MDA-MB-468, SKBR-3, MDA-MB-231, MDA-MB-436, MDA-MB-453, MDA-MB-157, MCF7, T47D, BT-549, ZR75-1, HCC202, and HCC1428) and found that the mean ALDEFLUOR-positive rate was 3.5% and positively correlated with ALDH1A1 protein expression^88^. The IHC results of tissue arrays of 481 breast cancer samples indicated that 26% of the samples expressed ALDH1A1, and ALDH1A1^+^ breast cancer cells accounted for approximately 5% of the total cells^24^. IHC analysis of another group containing 69 breast cancer samples yielded similar results, showing that 20.3% (14/69) of the samples expressed ALDH1A1, and breast cancer cells strongly positive for ALDH1A1 represented only 4.3% of the 14 positive samples^88^. The above ALDH1A1 positivity rates are consistent with the fact that CSCs account for only a small fraction of cancer cells^24^. Therefore, the researchers concluded that ALDH1A1 was the dominant enzyme subtype in the ALDEFLUOR^+^ population. However, recent studies have suggested that in addition to ALDH1A1, other ALDH subtypes, especially ALDH1A3, might also determine the positive rate of ALDEFLUOR in breast cancer^66^^,^^90^. In breast cancer cell lines with high ALDEFLUOR activity, such as MDA-MB-468 cells, ALDH1A3 knockdown significantly decreased the ALDEFLUOR-positive rate, whereas ALDH1A1 knockdown did not have this effect^90^. ALDH1A3 overexpression significantly increased ALDEFLUOR activity in MDA-MB-231 breast cancer cells, and the increase was greater than that caused by ALDH1A1 overexpression^91^. Marcato et al. analysed 4 cases of breast cancer samples and found that only 1 sample in the ALDEFLUOR^+^ subpopulation displayed high ALDH1A1 expression^90^. Correlation analysis of ALDH subtype expression with the ALDEFLUOR-positive rate in 7 breast cancer cell lines revealed higher ALDEFLUOR-positive rates in MDA-MB-468, SKBR3, and MDA-MB-435 cells^90^. ALDH1A1 knockdown in MAD-MB-435 cells only slightly affected the ALDEFLUOR-positive rate, whereas ALDH1A3 knockdown triggered a significant decrease (from 84.7% ± 0.95% to 36.9% ± 18.36%)^90^. The above results indicate that the ALDEFLUOR activity of some breast cancer cell lines and specimens from breast cancer patients may be mainly determined by ALDH1A3^90^.

To determine which ALDH isoforms play a decisive role in ALDEFLUOR activity, Zhou et al.^63^ cloned all 19 ALDH isoforms into lentiviral vectors and then established stable breast cancer SUM159 and MDA-MB-231 overexpression cell lines^63^. These 2 cell lines had relatively low endogenous levels of most ALDH isoforms and a low percentage of ALDH^+^ in the ALDEFLUOR assay^63^. As a result, 9 of the 19 ALDH isoforms may be involved in the level of ALDEFLUOR activity, including ALDH1A1, ALDH1A2, ALDH1A3, ALDH1B1, ALDH2, ALDH3A1, ALDH3A2, ALDH3B1, and ALDH5A1^63^. Upon stable knockdown of ALDH1A1, ALDH3, and ALDH3A2 in BT-474 cells and ALDH1A3, ALDH3A2, and ALDH3B1 in the MCF10A cell line, the ALDH^+^ ratio was measured^63^. The knockdown of ALDH2 in BT-474 cells and ALDH1A3 and ALDH3A2 in MCF10A cells significantly reduced the ALDH^+^ ratio, indicating that ALDH2 is a major player in the ALDEFLUOR activity of BT-474 cells, while ALDH1A3 and ALDH3A2 play an important role in the ALDEFLUOR activity of MCF10A cells^63^.

### Digestive tract

#### Oral mucosa

ALDEFLUOR activity in oral mucosa keratinocytes is primarily determined by ALDH1A3 and ALDH3A1^92^. The ALDEFLUOR-positive rate in normal oral mucosa cells is 14.1–17.6% (17.6% ± 7.4% in cells of fresh specimens and 14.1% ± 7.4% in cultured cells). IHC analysis revealed that the mucosal layer expressed ALDH1A3 and ALDH3A1 but not ALDH1A1^92^. ALDH1A3 expression gradually increased from the lower suprabasal layer to the upper suprabasal layer. ALDH3A1 is mainly expressed in the cytoplasm in the lower suprabasal layer cells and the nuclei in basal layer cells^92^. In situ hybridisation of mRNA confirmed the expression patterns of *ALDH1A3* and *ALDH3A1* in the oral mucosa^92^. Inhibition of ALDH1A3 or ALDH3A1 expression significantly decreased the ALDEFLUOR-positive rate, and the effect of ALDH1A3 intervention was more robust^92^. These results indicated that the ALDEFLUOR-positive rate in oral mucosa keratinocytes was determined by both ALDH1A3 and ALDH3A1 but predominantly by ALDH1A3^92^. Hedberg et al. reached a similar conclusion by analysing the gene expression profiles of cultured primary keratinocytes, the immortalised cell line SVpgC2a, and the buccal carcinoma cell line SqCC/Y1. The results indicated that all 3 types of cells expressed ALDH1A3 but not ALDH1A1, and ALDH3A1 was only expressed in the buccal carcinoma cell line^93^. Furthermore, the other enzyme subtypes ALDH3A2, ALDH4A1, ALDH7A1, and ALDH9A1 also displayed differential expression in the 3 types of cells^93^.

#### Colorectal cancer

Most researchers consider ALDH1A1 to be the dominant subtype determining ALDEFLUOR activity in colorectal cancer cells, and this enzyme subtype may be used as a marker for colorectal CSCs^32^^,^^33^. The mean ALDEFLUOR-positive rate in 3 colorectal cancer cell lines (LoVo, BE, and HT-29) was 15.5%, and ALDH1A1 displayed high expression in the ALDEFLUOR-positive subpopulation^88^. In addition, ALDEFLUOR^+^ cells isolated from colorectal cancer tissues had stem cell characteristics and could develop primary tumours in immunodeficient animals, while ALDEFLUOR^–^ cells could not^33^. IHC analysis indicated that colorectal cancer tissues had a positive rate of 94% (63/67) for ALDH1A1 expression^88^. Tumour cells with strong positive ALDH1A1 expression accounted for approximately 38.8% of the total cells and were localised in crypts, showing a spatial distribution similar to that of normal stem cells^88^. ALDH1A1 silencing decreased the tumorigenicity of HT-29 cells in athymic mice^34^. Therefore, these findings indicated that ALDH1A1 can be used as a colorectal cancer stem/progenitor cell marker and that it is the major enzyme subtype that determines ALDEFLUOR activity.

However, studies have demonstrated that ALDH isoforms are different in different colorectal cancer cell lines. HT-29 cells mainly express ALDH1A1 isoforms, HCT-116 cells mainly express ALDH1A3 isoforms, and LS-180 cells express ALDH1A1 and ALDH2 isoforms^34^.

Chen et al. reported that in addition to ALDH1A1, slim columnar cells in undifferentiated normal colon tissue also express a small amount of ALDH1B1^65^. ALDH1B1 is predominantly expressed in cells near the sides of the crypt bottom and is sporadically expressed in cells on both sides of the upper portion of the crypt, while no expression is evident in differentiated cells or stromal cells^65^. This distribution is highly similar to that of stem cells^65^. A total of 97.5% of colonic adenocarcinoma samples expressed high levels of ALDH1B1, whereas only 36.6% of samples expressed ALDH1A1, which was comparatively low. Thus, ALDH1B1 may represent another marker for colorectal CSCs and the ALDEFLUOR activity-determining enzyme subtype^65^.

### Pancreatic cancer

ALDEFLUOR^+^ cells account for approximately 3% of cells in pancreatic cancer and display CSCs characteristics^43–45^. Two groups of researchers have reported the measurement of ALDH1A1 expression in pancreatic cancer samples and showed similar positive rates of approximately 75%^94^. In positive samples, ALDH1A1^+^ cells accounted for more than 50% of the total tumour cells in 47% of the samples^94^. Therefore, the researchers concluded that ALDH1A1 is the major ALDH subtype that determines ALDEFLUOR activity in pancreatic cancer samples.

While the ALDEFLUOR-positive rates in the pancreatic cancer cell lines CAPAN-1, DAN-G, Panc1, and L3.6pl are 2.4–8.5%^44^, which is equivalent to those found in pancreatic cancer tissues, a comparison of 6 pancreatic cancer cell lines (BxPC3, T3M4, PANC1, SU8686, Colo-357, and AsPC-1) and 1 noncancerous pancreatic cell line (ACBRI) revealed a differential expression pattern for *ALDH1A1* mRNA^94^. Of these, BxPC3, T3M4, and PANC1 showed little or no *ALDH1A1* mRNA expression, and SU8686 and Colo-357 cells exhibited relatively low expression compared with ACBRI cells^94^. In addition, gene expression profiling of 3 pancreatic cancer cell lines indicated that AsPC-1 and BxPC3 cells primarily expressed ALDH1A3, whereas the expression of ALDH1A1 and ALDH1A3 in MIA PaCa cells was equivalent^95^. These results indicate that ALDEFLUOR activity in pancreatic cancer cells may not be solely determined by ALDH1A1.

### Lung cancer

The ALDEFLUOR-positive subpopulation derived from non-small cell lung cancer (NSCLC) cell lines was demonstrated to be highly clonogenic, tumorigenic, and invasive and displayed resistance to chemotherapeutic drugs. Moreover, ALDH1A1 expression was associated with poor prognosis in early-stage NSCLC^37^. Knockdown of ALDH1A1 and ALDH3A1 can inhibit clonogenicity and motility^96^ and induce sensitivity to 4-HC in NSCLC cell lines^97^. ALDH1A1 expression has been reported to be positively associated with Notch transcription, and pharmacologic and genetic interference with Notch can result in decreased ALDH1A1 activity in lung CSCs^98^^,^^99^. In addition, mechanistic investigations have identified that NFATc2/SOX2 coupling can upregulate ALDH1A1 expression by binding to its 5′ enhancer, attenuate oxidative stress induced by cancer drug treatment, and lead to increased resistance to chemotherapy and targeted therapy^100^. The embryonic transcription factor SOX9 was reported to promote stem-like properties and induce chemo resistance of NSCLC cells by trans activating ALDH1A1 expression^101^. ALDH1A1 knockdown was suggested to inhibit the invasive ability and in vivo tumorigenicity of ALDEFLUOR^+^ cells^38^, indicating that ALDH1A1 was the major subtype determining ALDEFLUOR activity in lung cancer. However, other studies have reported that ALDH1A3 might be the major enzyme subtype that determines ALDEFLUOR activity. The ALDEFLUOR-positive rates in Calu-1, H358, H1993, H2009, H2087, HCC44, HCC95, and HCC827 cells were 1–15%, and ALDEFLUOR^+^ cells exhibited relatively high ALDH1A3 expression in all cell lines^102^. ALDH1A3 knockdown decreased the ALDEFLUOR-positive rate and suppressed the colony formation ability and tumorigenicity of ALDEFLUOR^+^ cells^102^. Furthermore, upon knockdown of ALDH3A1 and ALDH1A1 in A549 cells, ALDEFLUOR-positive cells decreased by 95%, along with attenuated colony formation and migration ability^39^.

#### Melanoma

The ALDH1A isoenzyme is expressed in melanoma. Erfani et al. detected the expression of ALDH1A1 in 12 melanoma tissue samples through IHC, of which 67% (8/12) had low expression of ALDH1A1 and 33% (4/12) had high expression of ALDH1A1^103^. Recent studies have demonstrated that ALDH1As play essential roles in regulating the proliferation, apoptosis, and chemoresistance of melanoma CSCs^104^ and might have therapeutic potential^105^. ALDEFLUOR-positive melanoma CSCs were associated with chemoresistance, and ALDH1A (ALDH1A1 and ALDH1A3) silencing could attenuate cell proliferation and induce apoptosis in ALDEFLUOR-positive melanoma CSCs *in vitro* and suppress melanoma tumorigenesis in vivo^104^. Studies have found that ALDEFLUOR^+^ cells accounted for approximately 2% of melanoma cells (0.46–4.39%)^55^, and ALDEFLUOR^+^ subpopulations were detected in 8/9 cases of fresh tumour samples and 8/10 cases of xenografted tumours, with positive rates ranging from 0.08 to 1.15%^104^. Microarray analysis of ALDEFLUOR^+^ and ALDEFLUOR^–^ cells isolated from xenografted tumours indicated that the expression of ALDH1A1 and ALDH1A3 was more than 15 times higher in the ALDEFLUOR^+^ subpopulation^104^. These results suggest that ALDEFLUOR activity in primary melanoma may be mainly determined by both ALDH1A1 and ALDH1A3. However, in ALDEFLUOR^+^ subpopulations isolated from the 4 melanoma cell lines 1205Lu, A375, WM239A, and HS294T, *ALDH1A3* mRNA expression was more than 200 times higher than that of *ALDH1A1*^104^. Knockdown of ALDH1A3 expression significantly decreased the ALDEFLUOR-positive rate in 1205Lu and A375 cells, suggesting a contributing role of ALDH1A3 in determining ALDEFLUOR activity^104^. Altogether, the reason for the differences in ALDH1A1 and ALDH1A3 expression between primary melanomas and melanoma cell lines was not clear.

### Prostate cancer

Studies have shown that ALDEFLUOR-positive cells of prostatic cancer cell lines were enriched for clonogenicity, tumorigenicity, and metastatic ability^106^, and high ALDH1A1 expression correlates with lower overall survival, Gleason score, and pathologic stage in patients with primary prostate cancer^49^^,^^106^. The Wnt pathway was reported to directly regulate ALDH1A1 expression through β-catenin/TCF-dependent transcription, and inhibition of the Wnt/β-catenin signalling pathway was demonstrated to suppress the viability of prostate cancer cells with relatively high ALDH1A1 expression^16^. Work by Li et al. indicated that ALDEFLUOR^+^ cells in the PC3 and LNCaP cell lines possess stem cell characteristics and that ALDH1A1 was the major enzyme subtype determining ALDEFLUOR activity and may be used as a marker for prostate CSCs^49^. IHC analysis of 18 normal tissues and 163 prostate cancer tissue sections revealed low ALDH1A1 expression in the basal cell layers of normal prostate tissues and that it coexisted with the stem cell marker CD44^49^. In tumour tissues, ALDH1A1 was highly expressed in secretory-type cancer epithelial cells and neuroendocrine tumour cells^49^. Kalantari et al. studied the expression of ALDH1A1 in 105 prostate cancer tissues, 21 high-grade prostatic intraepithelial neoplasia tissues, and 31 benign prostatic hyperplasia tissues. In tumour tissues, 66% (69/105) had low expression, and 34% (36/105) had high expression^107^. There was a significant positive correlation between ALDH1A1 expression and the Gleason score. However, almost all BPH and HGPIN cases were not stained, and only 3% (1/31) of BPH cases showed strong staining^107^. However, van den Hoogen et al. found that the high ALDEFLUOR activity found in prostate cancer cells did not correlate with ALDH1A1 expression and that ALDH7A1 was the major enzyme subtype determining ALDEFLUOR activity^106^. Analysis of 8 prostate cancer cell lines (PC-3luc, PC-3M-Pro4lucA6, PC-3M-Pro4lucBIII, LNCaP, C4, C4-2, C4-2B, and DU145) and 6 primary prostate cancer specimens indicated that the ALDEFLUOR-positive rates were approximately 0.8–31% in prostate cancer cell lines and 0.5–12.5% in prostate cancer specimens^106^. The ALDH subtypes with higher expression levels included ALDH3A2, ALDH4A1, ALDH7A1, ALDH9A1, and ALDH18A1. High ALDH7A1 expression was observed in all primary cultured cells and in the PC-3, PC-3M-Pro4lucBIII, and DU145 cell lines^106^. ALDH7A1 knockdown was found to decrease ALDEFLUOR activity by 21%^50^. IHC analysis further confirmed the dominant expression of ALDH7A1 in prostate cancer^106^. In addition, ALDH4A1 and ALDH9A1 havebeen shown to be the major enzyme subtypes accounting for ALDEFLUOR activity in prostate cancer tissues, while the dominant enzyme subtypes in nontumor tissues and high-grade prostate intraepithelial neoplasia are ALDH3A2and ALDH18A1^106^.

### Head and neck squamous cell carcinoma (HNSCC)

In HNSCC samples, the mean ALDEFLUOR-positive rate was 3.5% (*n* = 6, 1.0–7.8%)^108^. ALDEFLUOR-positive HNSCC cells had stem cell characteristics with high CD44 expression and could form primary tumours in immunodeficient animals^35^^,^^108^. The mean ALDEFLUOR-positive rate in Fanconi anemia–head and neck squamous cell carcinoma (FA–HNSCC) cells was approximately 23% (VU-1365, 31% ± 2.9%; VU-1131, 23% ± 2.7%; OHSU-974, 15% ± 2.0%), higher than that in the UMSCC-22A HNSCC cell line (10.33% ± 3.0%)^109^. More than 10% of the tumour cells in HNSCC tumour samples expressed ALDH1A1, which was scattered as single cells or small groups of cells, whereas more than 25% of the cells expressed ALDH1A1, which was distributed as sheets or islands in FA–HNSCC tumour tissue samples^109^. IHC analysis of FA–HNSCC xenografted tumours yielded similar results^109^. Therefore, ALDH1A1 is considered the major subtype that determines ALDEFLUOR activity in HNSCC cells.

Nevertheless, other researchers argued that ALDEFLUOR activity in HNSCC is at least partially determined by ALDH1A3^110^. ALDH1A3 exhibited relatively high expression in FaDu and Cal33 cells compared to ALDH1A1^110^. Analysis of xenografted tumours derived from ALDEFLUOR-positive cells also showed high ALDH1A3 expression^110^. In other words, ALDH1A3 might also influence ALDEFLUOR activity^110^.

### Liver cancer

ALDH1A1 has been reported to be the dominant enzyme subtype determining ALDEFLUOR activity in hepatocellular cancer (HCC) and hepatoblastoma and was a differentially expressed protein in CD133^+^ and CD133^–^ cells of the Huh7 and PLC8024 HCC cell lines^41^. The ALDEFLUOR-positive rate was 55% in Hep3B cells and 1–8% in Huh7, H2M, PLC8024, and Hep2B cells, consistent with the theory that CSCs account for only a very small percentage of tumour cells. Therefore, researchers concluded that ALDH1A1 is the dominant enzyme subtype that determines ALDEFLUOR activity in HCC^41^. However, researchers observed no appreciable change in the HCC CSCs marker EpCAM and proliferation and sphere formation ability upon ALDH1A1 knockdown in Huh1 and Huh7 cells^111^. Moreover, patients with high ALDH1A1 expression had a higher degree of tumour differentiation. Thus, ALDH1A1 might otherwise be a differentiation marker with little relevance to the maintenance of stem cell characteristics of HCC^111^. RT–PCR analysis of 60 HCC samples and 47 paired HCC and adjacent nontumorous tissues revealed that *ALDH1A1* mRNA expression was not significantly different in tumour and nontumor tissues (1.36 ± 1.26 vs. 1.00 ± 0.53, *p* = 0.8858)^42^. IHC analysis also indicated that ALDH1A1 was not coexpressed with stem cell markers such as BMI1, EpCAM, CD13, CD24, CD90, and CD133^42^.

The major enzyme subtype that determines ALDEFLUOR activity was shown to be ALDH1A3 in intrahepatic CHOL^40^. The ALDEFLUOR-positive rates in the intrahepatic CHOLcell lines HuCCT1 and SNU1079 were 9.67% and 20.32%, respectively. The ALDEFLUOR^+^ cell population had stem cell characteristics, with higher resistance to gemcitabine than ALDEFLUOR^–^ cells^40^. Analysis of the mRNA expression of 19 ALDH subtypes in the ALDEFLUOR^+^ and ALDEFLUOR^–^ subpopulations indicated higher *ALDH1A3* and *ALDH1L1* expression in the ALDEFLUOR^+^ subpopulation of HuCCT1 cells and higher expression of *ALDH1A3*, *ALDH1B1*, *ALDH6A1*, *ALDH1A1*, *ALDH18A1*, *ALDH3B2*, and *ALDH3B1* in the ALDEFLUOR^+^ subpopulation of SUN1079 cells^40^. The ALDH subtype with the greatest abundance in the ALDEFLUOR^+^ populations in these 2 cell lines was *ALDH1A3*^40^. ALDH1A3 knockdown markedly reduced not only the invasion/migration ability but also the sensitivity to gemcitabine^40^. Altogether, these findings indicate that ALDH1A3 is the major enzyme subtype that determines ALDEFLUOR activity in CHOL.

### Brain tumours

Constitutive activation of the Notch signalling pathway was reported to rescue neurosphere differentiation of glioblastoma (GBM) in an RA-dependent process, indicating a functional role of ALDH in GBM tumorigenesis^112^. Some researchers consider ALDH1A1 to be the dominant enzyme subtype that determines ALDEFLUOR activity in glioma, and this subtype can be used as a marker of glioma stem cells (GSCs)^113^. Flow cytometry analysis of U251 cells and 1 case of primary cultured cells revealed that the ALDEFLUOR-positive rates were 6.9% ± 2.1% and 4.2% ± 1.9%, respectively, and the ALDEFLUOR population had CSC characteristics^113^. RT–PCR analysis of ALDEFLUOR^+^ and ALDEFLUOR^–^ subpopulations in U251 cells showed that *ALDH1A1* expression was significantly higher in the ALDEFLUOR^+^ subpopulation and was the dominant enzyme subtype for ALDEFLUOR. IHC analysis of 237 glioma samples revealed higher ALDH1A1 expression in high-grade gliomas (WHO III-IV) than in low-grade gliomas (WHO I-II)^113^. In addition, ALDH1A1 was the dominant subtype in the classical subtype of GBM, with 84.6% of the classical samples (*n* = 13) exhibiting high ALDH1A1 expression, while the ALDH1A1-positive rates were 12.5% and 21.7% in the proneural (PN) subtype (*n* = 36) and the other 2 subtypes (neural and mesenchymal (Mes)), respectively^113^.

In contrast, the results of another study suggested that ALDH1A3 might be the dominant subtype that determines ALDEFLUOR activity in glioma. IHC analysis revealed that 15 samples of normal tissues and 7 low-grade gliomas (WHO II) did not express ALDH1A3 and that 37 of 38 GBM cases expressed ALDH1A3^114^. In addition, Mes GSCs displayed relatively high invasiveness and intracranial tumorigenicity compared to PN GSCs, and the ALDEFLUOR activity of Mes GSCs was approximately 8 times higher than that of PN GSCs^114^. RT–PCR analysis of 19 ALDH subtypes indicated that *ALDH1A3* had significantly higher expression in Mes GSCs, with an approximately 150-fold higher expression level than that in PN GSCs^114^, whereas the other 18 enzyme subtypes were expressed at a very low level in both groups of GSCs^114^. Transcriptome microarray analysis also revealed 150-fold higher expression of ALDH1A3 in Mes GSCs than in PN GSCs^114^. Moreover, inhibition of ALDH1A3 could sufficiently attenuate the proliferation, sphere formation ability, and tumorigenicity of Mes GSCs^114^. These results indicate that ALDH1A3 is the dominant enzyme subtype that determines ALDEFLUOR activity in Mes GSCs.

The expression of different ALDH isoforms and their subcellular location in various cancer types were summarised in Table 1.

**Table 1.** Different ALDH isoforms and their location in various cancer types.

Cancer types	ALDH isoform	Location	References
AML	ALDH1A1, ALDH3A1		67,71,72
	ALDH1A1		70,73–75
K562 cell line	ALDH1, ALDH3, ALDH5, ALDH6, ALDH7, and ALDH8		17
Breast cancer	ALDH1A1	Cytoplasmic/membranous	24,83,84,88
	ALDH1A3	Nuclear/cytoplasmic/membranous	66,86,90,91
	ALDH1A1, ALDH1A3	Cytoplasmic/membranous Nuclear/cytoplasmic/membranous	87
MDA-MB-435, MDA-MB-468 cells	ALDH1A3		66,90
			
BT-474 cells	ALDH1A1 ALDH2		63,88
MDA-MB-468, MDA-MB-231, SKBR-3 cells	ALDH1A1 ALDH1A3		66,88
BT-483, BT-20, MDA-MB-231, MDA-MB-436, MDA-MB-453, MDA-MB-157, MCF7, T47D, BT-549, ZR75-1, HCC202 and HCC1428 cells	ALDH1A1		88
MCF10A cells	ALDH1A3, ALDH3A2		63
Buccal carcinoma cell line SqCC/Y1	ALDH1A3, ALDH3A1, ALDH3A2, ALDH4A1, ALDH7A1, ALDH9A1		93
Colorectal cancer	ALDH1A1	Cytoplasmic/membranous	32–34,88
	ALDH1B1 ALDH1A1	Cytoplasmic/membranous Cytoplasmic/membranous	65
HCT-116 cells	ALDH1A3		34
LoVo, and BE cells	ALDH1A1		88
HT-29 cells	ALDH1A1		34,88
LS-180 cells	ALDH1A1 ALDH2		34
Pancreatic cancer	ALDH1A1	Cytoplasmic/membranous	43,94
AsPC-1, BxPC3	ALDH1A3		95
MIA PaCa cells	ALDH1A1 ALDH1A3		95
NSCLC	ALDH1A1	Cytoplasmic/membranous	37,38,98–101
	ALDH1A1 ALDH3A1	Cytoplasmic/membranous Cytoplasmic/membranous/nuclear	39,96,97
A549 cells	ALDH1A1		39
Calu-1, H358, H1993, H2009, H2087, HCC44, HCC95, and HCC827 cells	ALDH1A3		102
Melanoma	ALDH1A1 ALDH1A3	Cytoplasmic/membranous Cytoplasmic/membranous	104,105
1205Lu, A375, WM239A, and HS294T	ALDH1A1 ALDH1A3		104
Prostate cancer	ALDH1A1	Cytoplasmic/membranous	16,49,106
	ALDH7A1, ALDH3A2, ALDH4A1, ALDH9A1,	Cytoplasmic/membranous/nuclear Cytoplasmic/membranous/nuclear Cytoplasmic/membranous Cytoplasmic/membranous	106
	ALDH18A1	Cytoplasmic/membranous	
	ALDH7A1	Cytoplasmic/membranous/nuclear	50
PC3 and LNCaP cells	ALDH1A1		49
HNSCC	ALDH1A1	Cytoplasmic/membranous	109
	ALDH1A3	Nuclear	110
FaDu and Cal33 cells	ALDH1A3		110
Liver cancer	ALDH1A1	Cytoplasmic/membranous	41,111
intrahepatic cholangiocarcinoma	ALDH1A3		40
HuCCT1 cells	ALDH1A3 ALDH1L1		
SUN1079 cells	ALDH1A3, ALDH1B1, ALDH6A1, ALDH1A1, ALDH18A1, ALDH3B2, ALDH3B1		
Hep3B, Huh7, H2M, PLC8024, Hep2B, Huh1 and Huh7 cell	ALDH1A1		111
Brain tumours			
Glioma	ALDH1A1	Cytoplasmic/membranous	113
Glioma	ALDH1A3	Nuclear	114
U251 cells	ALDH1A1		113

### Articular chondrocyte stem cells

Studies of human articular chondrocyte stem cells demonstrated that ALDH1A2, ALDH1A3, and ALDH2 are strongly expressed in chondrocytes, whereas ALDH1A1 expression is low. In isolated ALDEFLUOR^+^ chondrocyte subpopulations, only ALDH1A2 and ALDH1A3 showed remarkably high expression^115^.

## Possible mechanisms underlying the ALDEFLUOR-positive rate and study directions

With the completion of in-depth studies and the availability of commercial antibodies specific for ALDH enzyme subtypes, accumulative efforts have begun to clarify the characteristics of the ALDEFLUOR^+^ cell population. In addition to ALDH1A1, other ALDH subtypes are now understood to also influence the ALDEFLUOR-positive rate, including ALDH1A2, ALDH1A3, ALDH1B1, ALDH3A2, ALDH4A1, ALDH7A1, and ALDH9A1. The possible reasons for this phenomenon are discussed below.

### BAAA is not the only substrate of ALDH1A1

In early studies, BAAA was generally considered a specific substrate of ALDH1A1, and the ALDEFLUOR-positive rate was thought to directly reflect the expression level and activity of ALDH1A1^116^. However, all members of the ALDH family have similar physiological functions and participate in the metabolism of hydrocarbons, biogenic amines, RA, and steroids and in lipid peroxidation^117^. With the exception of ALDH6A1, which uses CoA as a cofactor, the other enzyme subtypes all use NAD(P^+^) as a cofactor, and their catalytic mechanisms in the dehydrogenase and lipase functions are similar and may be associated with the highly conserved catalytic residues Cys302, Lys192, and Glu268^118^. The substrates of each isozyme also have similar structures^117^. Several isozymes (1A1, 1A2, 1A3, 2, 3A1, 7A1, and 8A1) in the ALDH family are able to utilise BAAA as a substrate^3^^,^^17^^,^^66^^,^^104^^,^^119^, suggesting that ALDEFLUOR^+^ cells might be a mixed population and that ALDEFLUOR activity is not simply determined by the expression level and activity of ALDH1A1^17^. When ALDH1A2 and ALDH2 were overexpressed in Beas-2b immortalised lung epithelial cells that did not contain an ALDEFLUOR^+^ cell subpopulation, the resulting Beas-2B^ALDH1A2^ or Beas-2B^ALDH2^ cells were mixed with K562 leukaemia cells or H1299 lung cancer cells at percentages of 20–25%, and the mixed cell populations were analysed using the ALDEFLUOR system^17^. The results indicated that the ALDEFLUOR-positive rates increased significantly in both mixed cell populations. These results indicate that in addition to ALDH1A1, the enzyme activities of ALDH1A2 and ALDH2 also contribute to activity in the ALDEFLUOR system^17^. However, although researchers now recognise that BAAA may be a common substrate of various ALDH subtypes, progress in identifying specific substrates for ALDH subtypes has been limited, and this topic should become a key research topic in the future.

### DEAB does not specifically inhibit ALDH1A1 activity and is a substrate for various ALDH subtypes

DEAB was previously considered a selective inhibitor of ALDH1A1^39^^,^^120^ and was often used as a negative control in ALDEFLUOR analysis (Figure 1). However, recent studies have found that DEAB is not a specific inhibitor of ALDH1A1 and that it has a stronger inhibitory effect (IC_50_ 0.057–15 μM) on other enzyme subtypes, including ALDH1A2, ALDH1A3, ALDH1B1, ALDH2, and ALDH5A1^17^^,^^63^^,^^64^^,^^90^. The order of sensitivity of ALDH subtypes to DEAB was ALDH1A1 < ALDH2 < ALDH1A2 < ALDH1B1  < ALDH1A3 < ALDH3A1 < ALDH5A1^63^^,^^64^. The order of the catalytic efficiency of different ALDH subtypes on DEAB, from strong to weak, was shown to be ALDH1A1 > ALDH1B1, ALDH1A3, ALDH5A1 > ALDH2^64^. Furthermore, DEAB had inhibitory effects on ALDH3A1 in oral mucosal epithelial cells^92^, and the *Vmax*/*K_m_* of ALDH3A1 on DEAB was even higher than that of its conventional substrate benzaldehyde^64^. These findings at least partially explain why increased DEAB concentration and increased incubation time may both influence the ALDEFLUOR-positive rate observed during practical application of the method. For example, in cell lines with higher ALDEFLUOR-positive rates, such as MDA-MB-468, SKBR3, and MDA-MB-435 cells, a reduction in the DEAB concentration can significantly increase the ALDEFLUOR-positive rate^66^. However, in cell lines with moderate ALDEFLUOR-positive rates, such as BT-20 cells, and in those with lower ALDEFLUOR-positive rates, such as MCF7, T47D, and MDA-MB-231 cells, the effects of using different concentrations of DEAB (15 μM and 100 μM) on ALDEFLUOR-positive rates are not as obvious^66^. Similarly, inhibition of ALDH1A2 and ALDH2 by DEAB showed an obvious concentration dependence in K562 leukaemia cells and H1299 lung cancer cells^17^. Therefore, exploration and verification of the optimal incubation time and the optimal concentration of DEAB for use in different cells is another desirable area for future research.

Ibrahim et al.^121^ investigated the inhibitory effect of DEAB analogs on different isoforms of ALDH, which were synthesised using either aliphatic or aromatic nucleophilic substitution in a one-step reaction. Analogs 18 (4-(dipropylamino)-3-nitrobenzaldehyde) and 19 (4-(diethylamino)-3-nitrobenzaldehyde) showed potent inhibitory activity against ALDH3A1^121^. Analogs 14 (3-bromo-4-(dipropylamino)benzaldehyde), 15 (3-methyl-4-(piperidin-1-yl)benzaldehyde), and 16 (4-isopropoxybenzaldehyde) had potent inhibitory activity against ALDH1A3^121^. Analog 14 showed the highest antiproliferative activity in the prostate cancer cell lines DU145 and PC3, with IC_50_ values of 61 and 47 μM, respectively, and analog 14 also showed very low activity as a substrate for ALDH1A1 and ALDH3A1 isoforms, demonstrating the advantage of its higher selectivity^121^. The specificity of the ALDEFLUOR assay was improved by using compounds 14 and 15 to label ALDH1A3-expressing cells or 18 and 19 for ALDH3A1-expressing cells and compared to the results from DEAB^121^ (Figure 1).

To date, specific inhibitors of most ALDH isozymes, including ALDH1A1, have not been systematically identified^118^. Nevertheless, natural and synthesised compounds have been developed, including disulphiram (Figure 1), which can irreversibly inhibit ALDH1A1 and ALDH2 at the same time and is clinically used to treat alcoholism^118^ and cocaine addiction^122^. In addition, the H2 receptor antagonist cimetidine, which is used in the treatment of peptic ulcers, has weaker non-competitive inhibitory effects on ALDH1A1 enzyme activity in the liver, small intestine, and stomach at therapeutic concentrations (0.015 mM) and competitive inhibitory effects on ALDH2 and ALDH3A1^123^. Therefore, the identification of specific inhibitors of each individual ALDH enzyme subtype should also be a key focus of future research^119^. Notably, the identification of specific inhibitors of individual ALDH subtypes will not only help confirm the specific isozyme determining ALDEFLUOR activity in cells but is also necessary for accurate intervention in all ALDH subtypes with different functions.

ALDH isozymes have a high degree of sequence homology, with ALDH1A1 sharing more than 70% sequence homology with ALDH1A2 and ALDH1A3^124–127^. The development of selective inhibitors for each ALDH isoform has been hampered by a high degree of sequence and structural homology. Li et al. used the structure of human ALDH1A combined with in silico modelling to identify a selective, active-site inhibitor of ALDH1A3, MCI-INI-3^124^ (Figure 1). The compound MCI-INI-3 is a potent selective inhibitor of recombinant human ALDH1A3, with greater than 140-fold selectivity for ALDH1A3 compared to the closely related isoform ALDH1A1^124^. NR6 is a new selective ALDH1A3 inhibitor^128^ (Figure 1). The crystal structure determined by X-ray analysis reveals that NR6 binds to a nonconserved tyrosine residue of ALDH1A3, which drives the selectivity for this isoform, which is supported by computational binding simulations^128^. NR6 has cytotoxic activity against glioblastoma and colorectal cancer cells^128^.

**Figure 2.**
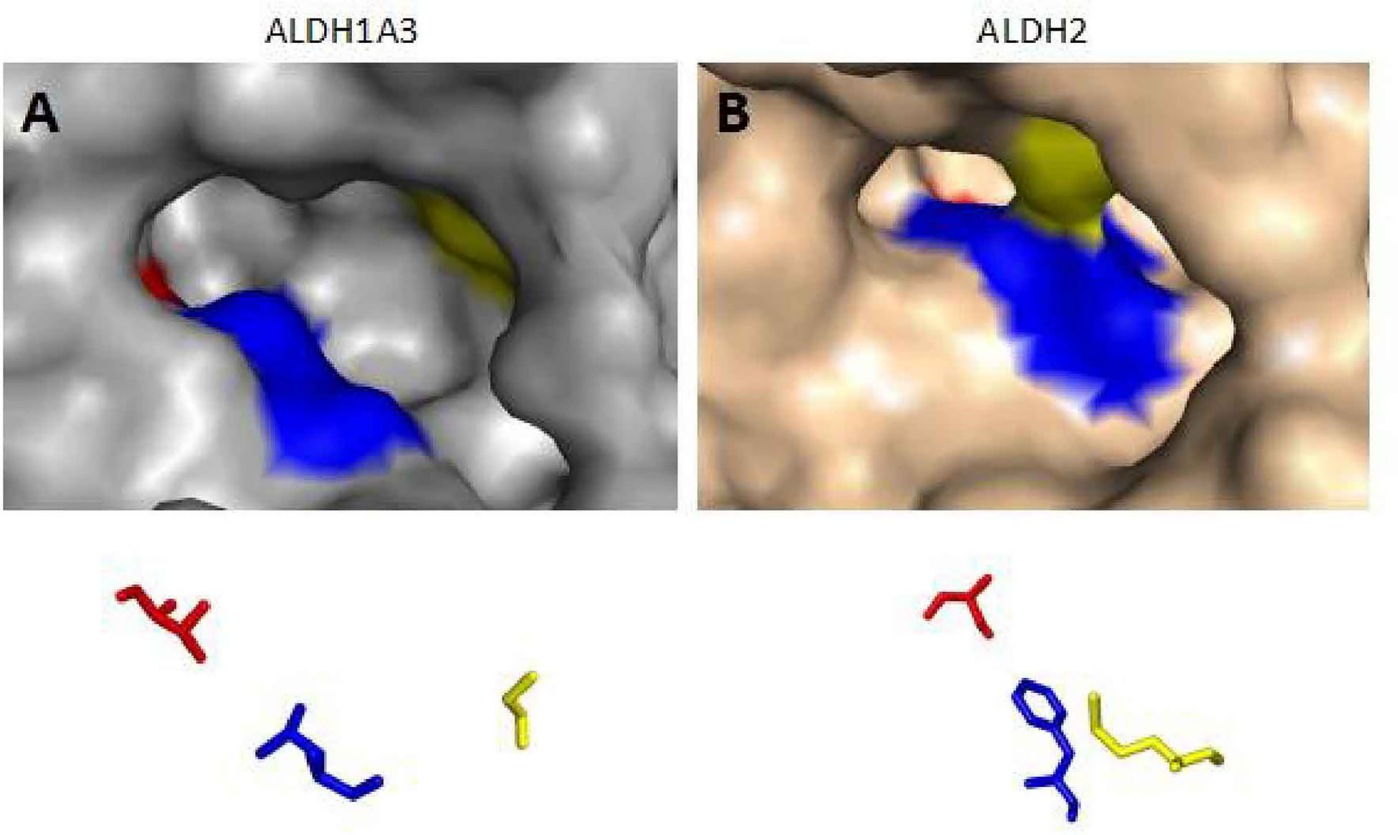
The substrate binding positions of ALDH1A3 and ALDH2. Surface representation of ALDH1A3 (A, in gray); the conserved ligand-binding residues are occupied by G136, L471, and T315 (PDB:5FHZ). Surface representation of human mitochondrial ALDH2 (B, in pink), with positions 124, 459, and 303 shown (PDB:1O01). The binding sites from the two monomers are shown as sticks and coloured red, blue, and yellow according to their positions.

Because the Rossmann fold structure at the NAD(P)^+^ cofactor-binding site of other dehydrogenase families, such as lactate dehydrogenase and alcohol dehydrogenase, is different from that of the ALDH family^129^, this structure is an important target for developing inhibitors for the ALDH family. Recently, Feng et al. discovered a selective imidazolium-based inhibitor of ALDH1B1 (half-maximum inhibitory concentration (IC_50_) = 57 nM)^130^. This inhibitor acted in a non-competitive manner with respect to the aldehyde substrate and exhibited an uncompetitive relationship with NAD^+^^130^. However, the Rossmann fold structures at the NAD(P)^+^ binding sites of all ALDH enzyme subtypes display high similarity. Further studies indicated that different aldehyde group binding sites have evolved in different subtypes in the ALDH family^117^^,^^131^. Therefore, Morgan et al. considered that targeting substrate binding sites or allosteric sites other than the active sites might be suitable for screening specific inhibitors^132^. Specifically, 3 topologically conserved residues located within the ligand-binding pocket control the substrate specificity of different ALDH subtypes, such as ALDH1A3 and ALDH2^133^ (Figure 2). Of course, the direct use of the high-throughput screening method offers another strategy for acquiring inhibitors for all ALDH subtypes. However, how to evaluate their inhibitory efficiency is also worth exploring. In the past, an ultraviolet light spectrophotometer was used to evaluate the inhibitory efficiency of candidate molecules on ALDH activity by measuring the intensity of the absorption peak of NADH at 340 nm. However, compounds in the library to be screened might have the same ultraviolet light absorption wavelength as NADH, which would reduce the accuracy of the results. ALDH family members also have esterase activity and can hydrolyse p-nitrophenyl acetate into nitrophenol, producing an absorption peak at 405 nm^132^. Therefore, in recent years, some researchers have also used the intensity of the absorption peak at 405 nm to evaluate the inhibition of ALDH by candidate molecules. For example, Morgan et al. adopted this strategy to identify a small molecule among 6,400 compounds, CM037 (Figure 1), that specifically inhibits ALDH1A1 with an IC_50_ of 4.6 ± 0.8 μM^132^. This compound significantly influenced the sphere formation ability of ovarian cancer cells^134^. Parajuli et al. combined these 2 strategies using a method that involved measuring the intensity of the absorption peaks at 340 nm and 405 nm to identify 14 small-molecule inhibitors that could better inhibit the dehydrogenase and esterase activities of ALDH3A1^135^. ABMM-15 and ABMM-16 are selective ALDH1A3 inhibitors designed based on the similarity of physiological substrates and are a class of compounds with a benzyloxybenzaldehyde scaffold^136^ (Figure 1). ALDH-positive A549 and ALDH-negative H1299 cells were used to validate the selectivity and cytotoxicity of the compounds against ALDH1A1, ALDH1A3, and ALDH3A1^136^. The 2 compounds were found to be the most potent and selective inhibitors of ALDH1A3^136^.

### Tumour type and tissue/cell origin are important influencing factors

As mentioned above, BAAA can be catalysed by various ALDH subtypes. One important question is whether differences in the expression of ALDH subtypes in various normal tissues and tumours result in differences in the dominant ALDH subtypes in cells isolated and acquired using the ALDEFLUOR system.

**Figure 3.**
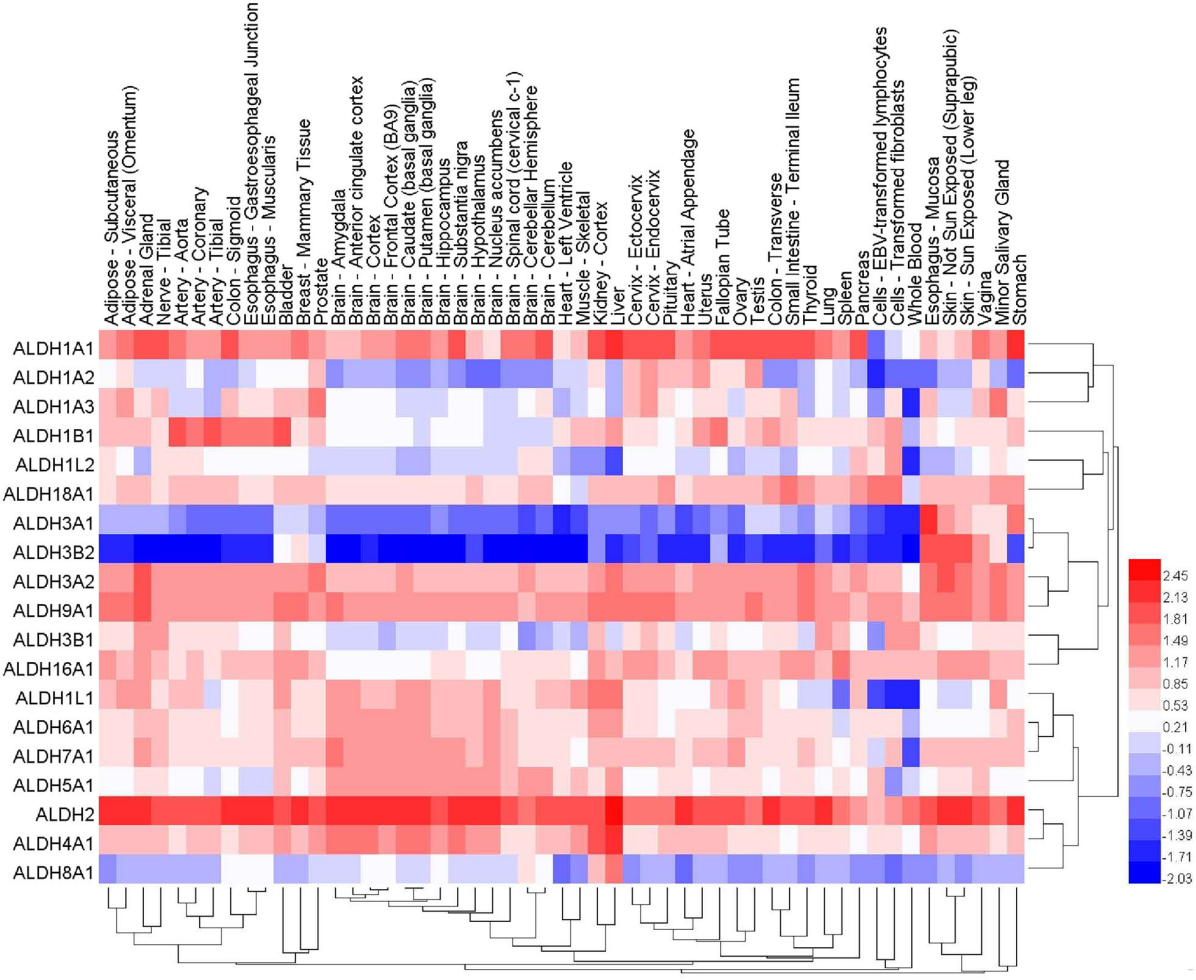
The expression levels of different ALDH family isoforms in 30 normal tissues. The data were derived from the GTEx (Genotype-Tissue Expression Project). Dendrograms and heat maps of ALDH mRNA expression in various normal tissues are shown.

We used public databases to analyse the mRNA expression of different ALDH subtypes in 30 normal tissues (Figure 3). The results indicated that except for blood, which contained ALDH3B1 as the dominant subtype, the normal oesophageal mucosa, which contained ALDH3A1 as the dominant subtype, and prostate and salivary glands, which contained ALDH1A3 as the dominant subtype, most tissues all had ALDH2 and ALDH1A1 as the dominant subtypes. These findings are consistent with the results showing that ALDH1A1 is predominantly expressed in the testis, brain, eye^137^, liver^138^, kidney epithelium, neural stem cells, and HSCs^139^^,^^140^, while ALDH1A3 is mainly expressed in the kidney, salivary glands, stomach^141^, foetal nasal mucosa^142^, and mammary glands^89^. Studies of buccal carcinoma^93^, HNSCC^110^, and breast cancer^66^ tissue also indicated that ALDH1A3 was the dominant subtype and that tumour cells with high ALDH1A3 expression had CSCs characteristics; this result is consistent with the high expression of ALDH1A3 in normal tissues of the same origin.

**Figure 4.**
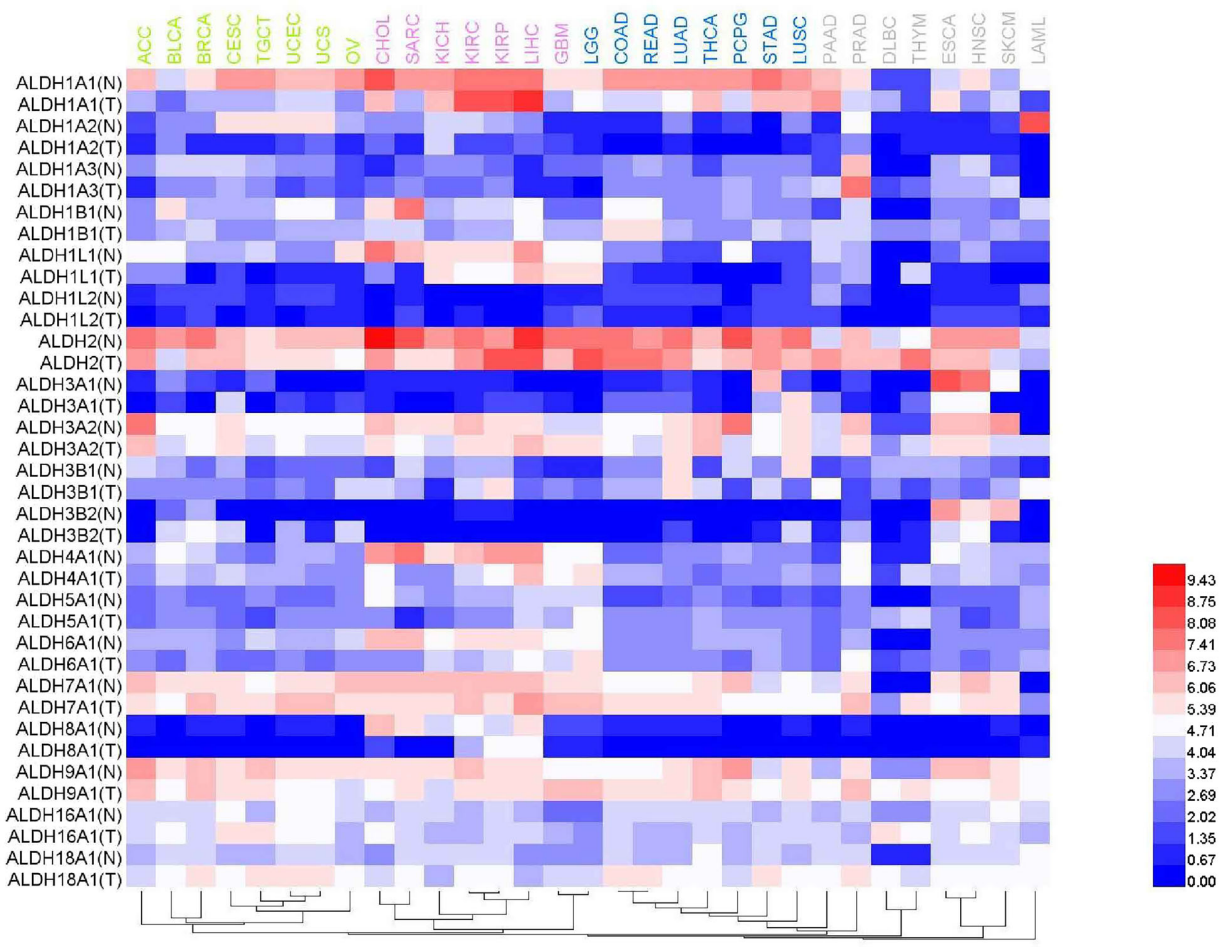
Comparison of the expression levels of different ALDH subtypes in various tumour tissues and corresponding normal tissues. A heat map was generated based on a TCGA dataset. The dendrogram of tumours (top) was divided into 4 parts using different colours.

Despite the foregoing information, researchers have reached inconsistent conclusions regarding some tissues and tumours. First, analysis of data in public databases indicated that the expression of ALDH3B1 in normal lung tissues was higher than that reported above. Some researchers demonstrated that the expression of ALDH3B1 in lung cancer was upregulated^143^, which is consistent with the trend in which some subtypes with higher expression in normal tissues were also highly expressed in tumours. However, some studies found that ALDH1A3 was the major subtype in NSCLC^102^. Second, ALDH1A1 was reported to be the dominant ALDH subtype in liver and brain tissues. Most researchers consider that ALDH1A1 is also the dominant ALDH subtype expressed in liver cancer^41^ and brain cancer^113^. However, some researchers reported that ALDH1A3 was the dominant subtype and that cells expressing ALDH1A3 had CSCs characteristics^40^. Other studies found that the dominant subtype in normal tissues was not dominant in tumours derived from those tissues. For example, the dominant subtype in blood was ALDH3B1; however, the expression of ALDH1A1, ALDH1A2, and ALDH1B1 in the K562 chronic myeloid leukaemia cell line was higher than that of ALDH3B1 and ALDH3A1^17^. Marchitti et al. reported that ALDH3B1 expression was upregulated in various tumours, including breast cancer, ovarian cancer, and colorectal cancer^143^. However, data in public databases indicate that the expression of ALDH3B1 in normal mammary, ovarian, and colonic tissues occurs at a lower level and that the dominant enzyme subtype in these tissues is ALDH1A1. Similarly, we also used public data resources to analyse the expression of different ALDH subtypes in various tumour tissues and corresponding paracancerous tissues (Figure 4). Hepatocellular carcinoma is a special case because the expression of many ALDH subtypes, including ALDH1A1, ALDH1L1, ALDH2, ALDH3A2, ALDH4A1, ALDH7A1, and ALDH9A1, is relatively high in this cancer. High expression of ALDH1L2, ALDH5A1, and ALDH6A1 can be observed in low-grade glioma. The results also revealed that ALDH1A2 expression is highest in mesothelioma (data not shown) and that the tumour type with the highest ALDH1A3 expression level is prostate cancer. In addition, the expression of ALDH3A2 in adenocarcinoma, ALDH7A1 in prostate cancer and glioma, and ALDH9A1 in breast cancer and thyroid cancer is also high. In general, the expression of ALDHs in tumour tissues is higher than that in corresponding paracancerous tissues. For example, ALDH1L1, ALDH4A1, ALDH6A1, and ALDH8A1 expression in sarcoma is lower than that in corresponding mesenchymal tissues. However, this trend is sometimes reversed in thymoma: ALDH1A1, ALDH1B1, ALDH3A2, ALDH5A1, ALDH6A1, ALDH7A1, ALDH9A1, and ALDH18A1 expression is higher in thymoma than in corresponding paracancerous tissues.

Interestingly, when tumours, primary specimens, and cell lines derived from the same tissues were compared, the dominant ALDH subtypes that determined the ALDEFLUOR-positive rate were found not to be the same. As previously mentioned, ALDEFLUOR activity in primary melanoma may be mainly determined by ALDH1A1 and ALDH1A3. However, in melanoma cell lines (1205Lu, A375, WM239A, and HS294T), ALDEFLUOR activity may be mainly determined by ALDH1A3. Determination of the reason for this discrepancy will require further in-depth studies.

## Clinical significance of ALDH subtypes in different cancers

The catalytic activity of ALDH can be used as a marker for the identification and isolation of tumour stem cells and is associated with prognosis in patients with a variety of tumour types^24^^,^^144^^,^^145^.

ALDH1A1, a marker of tumour stem cells in breast, prostate, colon, and lung cancers, is a cytoplasmic enzyme that is upregulated in tumour cells; ALDH1A1 is associated with poor prognosis of many tumours (breast cancer, melanoma, etc.) and has an important role in promoting tumour angiogenesis and metastasis and in acquiring resistance to anticancer drugs^146–148^.

Low ALDH1A2 expression is associated with poor prognosis and shorter disease-free and overall survival for ovarian cancer patients. ALDH1A2 suppresses the proliferation and migration of epithelial ovarian cancer cells by downregulating STAT3, and enhanced ALDH1A2-related signalling may provide new opportunities for therapeutic intervention in ovarian cancer^149^.

ALDH1A3 activates the PI3K/AKT/mTOR signalling pathway and its downstream target PPARγ, leading to increased expression of HK2, which in turn increases glycolysis in pancreatic ductal carcinoma cells, thereby promoting tumour metastasis^150^. CHOL cells with high ALDH1A3 levels not only migrated more quickly but were more resistant to gemcitabine^40^. In addition, knockdown of ALDH1A3 expression in HuCCT1 cells markedly reduced not only their sensitivity to gemcitabine, which might be attributed to a decreased expression of ribonucleotide reductase M1, but also their migration^40^. ALDH1A3 plays an important role in enhancing the malignant behaviour of CHOL and serves as a new therapeutic target^40^.

Our previous studies in colorectal cancer have demonstrated that ALDH1A3 can promote the invasion and metastasis of colorectal cancer cells through the miR-200-ZEB1/SANI2 axis and that inhibition of ALDH1A3 with the identified compound YD1701 may be an effective therapeutic approach to prevent colorectal cancer metastasis^151^. Other investigators have reported that knockdown of ALDH1B1 in colorectal cancer SW480 cells downregulates Wnt, Notch, and PI3K/Akt signalling and that the growth of 5-fluorouracil-resistant colorectal cancer spheroids and patient-derived organoids can be inhibited using small-molecule inhibitors of ALDH1B1^130^^,^^152^.

Studies have demonstrated that high expression of ALDH3B2 in perihilar CHOL is associated with higher tumour T stage and M stage and a higher incidence of neuroinvasion. High ALDH3B2 expression was an independent prognostic factor for intrahepatic CHOL and distal CHOL^153^. Inhibition of ALDH3B2 expression may inhibit the proliferation and clonogenic ability of CHOL by inducing G1 phase arrest. ALDH3B2 may also promote CHOL progression by promoting epithelial–mesenchymal transition^153^. Targeted inhibition of ALDH3B2 function is expected to inhibit the metastasis of CHOL cells and prolong the survival of patients^153^.

The expression of ALDH18A1 was significantly associated with the overall survival of neuroblastoma patients^154^. On the one hand, ALDH18A1 positively regulates MYCN gene expression at the transcriptional and posttranscriptional levels through the miR-29b/SP1 self-regulatory loop and miRNA regulatory network, respectively, which in turn affects the division and proliferation, self-renewal, and tumorigenic capacity of neuroblastoma cells^154^. On the other hand, the transcription factor N-Myc can directly target the ALDH18A1 promoter through specific binding sites to activate ALDH18A1 transcription and increase ALDH18A1 expression levels^154^. Administration of the ALDH18A1-specific inhibitor YG1702 inhibits N-Myc expression and attenuates the growth of neuroblastoma cells^154^.

## Clinical trials on ALDH in cancers

Targeting CSCs has great prospects for preventing metastasis and reducing the risk of drug resistance and recurrence. The vast majority of patients with AML achieve complete remission after standard induction chemotherapy. However, most patients die from tumour recurrence. LSCs are more resistant to chemotherapy and are a major cause of relapse^79^. DIMATE is an active enzyme-dependent, competitive, irreversible, broad spectrum inhibitor of ALDHs 1s and 3s^155^. A clinical study was proposed to test the *in vitro* sensitivity of leukaemia-derived CD4/CD38 cells to the DIMATE compound and to analyse the effect of DIMATE on the parameters of proliferation, apoptosis, secretory cytokines, and transcriptomic changesto better define the therapeutic index of the compound. HSC from healthy donors were used as controls^155^. The study did not provide results^155^ (Table 2). Venton et al. reported that in cultured cells, DIMATE was a potent inhibitor of ALDH 1 s and ALDH 3 s and had major cytotoxic activity against human AML cell lines^79^. In addition, DIMATE was selectively cytotoxic in a leukemic population enriched in LSCs, but unlike conventional chemotherapy, DIMATE was not toxic to healthy HSC^79^.

**Table 2.** Clinical trials on ALDH in cancers.

Description	Status	Conditions	Phase	Study Results	Trial identifier
Evaluation of the effectiveness of an aldehyde inhibitor Dehydrogenases (DIMATE) on the cell population of leukemic or normal stem cells	Recruiting	Leukaemia	Not Applicable	No Results Available	NCT02748850
Association of ALDH with disease incidence	Not yet recruiting	Oral Squamous Cell Carcinoma	Not Applicable	No Results Available	NCT04270201
CSCs marker	Completed	Breast Cancer	Not Applicable	No Results Available	NCT00949013
CSCs marker	Completed	Breast Cancer	II	Has Results	NCT01190345
CSCs marker	Completed	Breast Cancer	II	No Results Available	NCT01688609
CSCs marker	Completed	Breast Cancer	Early Phase 1	No Results Available	NCT04142892
CSCs marker	Completed	Healthy Lobular Breast Carcinoma in Situ	I	No Results Available	NCT01077453
CSCs marker	Completed	Breast Cancer	II, III	No Results Available	NCT01426880
CSCs marker	Completed	Lung Cancer, Non-small Cell Relapse/Recurrence CSC	Not Applicable	No Results Available	NCT04634630
CSCs marker	Completed	Lymphoma	II	Has Results	NCT00369681
CSCs marker	Completed	Ovarian Neoplasms, Fallopian Tube Neoplasms, Peritoneal Neoplasms	I	No Results Available	NCT02903771
CSCs marker	Completed	Pancreatic Cancer	I, II	No Results Available	NCT03410030

A forthcoming case–control study of OSCC patients and healthy volunteers will collect saliva samples from both groups and submit them for sequencing analysis to evaluate the frequency of ALDH1b1 and ALDH2 polymorphisms in the Brazilian population and correlate OSCC risk to alcohol consumption or smoking through applied questionnaires. These data will help characterise genetic variants of ALDH1B1 and ALDH2 in the Brazilian population and support the development of future public policies to reduce the main risk factors for OSCC, especially in those with these genetic variants (NCT04270201) (Table 2).

In addition, a few clinical trials are evaluating the effectiveness of treatment on the expression of CSC markers, mainly measuring the activity of ALDH, including breast cancer (clinical trial numbers: NCT00949013, NCT01190345, NCT01688609, NCT04142892, NCT01077453, NCT01426880), lung cancer (NCT04634630), lymphoma (NCT00369681), gynecological tumours (NCT02903771), and prostate cancer (NCT03410030) (Table 2).

## Conclusions

In this review, we summarise the predominant enzymes and groups of enzymes among the 19 members of the ALDH family that determine the positive rate of ALDEFLUOR in different normal tissues and tumours. In addition to ALDH1A1, other ALDH isoforms, such as ALDH1A3, ALDH2, and ALDH3A1, should also be considered in ALDEFLUOR analyses.

Although the ALDEFLUOR system provides a powerful tool for in-depth study of the characteristics of stem cells and cancers, this system has some limitations due to the specificity of the fluorescent substrate BAAA and the control inhibitor DEAB. Future research should focus on the development of new systems based on cell-type-specific substrates and inhibitors.

## Data Availability

The materials that support the conclusions of this review have been included within the article.
